# Nomograms for prediction of breast cancer in breast imaging reporting and data system (BI-RADS) ultrasound category 4 or 5 lesions: A single-center retrospective study based on radiomics features

**DOI:** 10.3389/fonc.2022.894476

**Published:** 2022-09-23

**Authors:** Zhi-Liang Hong, Sheng Chen, Xiao-Rui Peng, Jian-Wei Li, Jian-Chuan Yang, Song-Song Wu

**Affiliations:** ^1^ Shengli Clinical Medical College of Fujian Medical University, Fuzhou, China; ^2^ Department of Ultrasound, Fujian Provincial Hospital, Fuzhou, China; ^3^ Clinical Skills Teaching Center, Fujian Medical University, Fuzhou, China

**Keywords:** breast cancer, radiomics, ultrasound, nomograms, BI-RADS

## Abstract

**Purpose:**

To develop nomograms for predicting breast malignancy in BI-RADS ultrasound (US) category 4 or 5 lesions based on radiomics features.

**Methods:**

Between January 2020 and January 2022, we prospectively collected and retrospectively analyzed the medical records of 496 patients pathologically proven breast lesions in our hospital. The data set was divided into model training group and validation testing group with a 75/25 split. Radiomics features were obtained using the PyRadiomics package, and the radiomics score was established by least absolute shrinkage and selection operator regression. A nomogram was developed for BI-RADS US category 4 or 5 lesions according to the results of multivariate regression analysis from the training group.

**Result:**

The AUCs of radiomics score consisting of 31 US features was 0.886. The AUC of the model constructed with radiomics score, patient age, lesion diameter identified by US and BI-RADS category involved was 0.956 (95% CI, 0.910–0.972) for the training group and 0.937 (95% CI, 0.893–0.965) for the validation cohort. The calibration curves showed good agreement between the predictions and observations.

**Conclusions:**

Both nomogram and radiomics score can be used as methods to assist radiologists and clinicians in predicting breast malignancy in BI-RADS US category 4 or 5 lesions.

## Highlights

1. Breast cancer, the malignant tumor with the highest incidence in women, has become the top killer of women’s health.
2. Traditional imaging examinations have limited value for evaluating the status of breast lesions in BI-RADS ultrasound category 4 or 5.
3. We developed a radiomic nomogram based on US imagings to predict breast malignancy in BI-RADS US category 4 or 5 lesions.

## Introduction

According to the statistics of the World Health Organization (WHO) (1), breast cancer(BC), the malignant tumor with the highest incidence in women, has become the top killer of women’s health. New cases of BC worldwide accounted for about a quarter of all malignancies annually (2). The incidence of BC in Chinese women is 41.82 per 100,000, and the fatality rate is 9.90 per 100,000.The climbing yearly incidence and younger trend have also been observed. Early detection, diagnosis and treatment is unquestionably crucial to the reduction of the morbidity and mortality related to BC.

In the second edition of the ACR BI-RADS US atlas, breast lesions are assigned a category after the analysis and judgment of their sonographic characteristics (3). There are seven categories in total (3). Among them, category 4 is defined as suspicious lesion with 5% to 95% malignant probability that is recommended for biopsy. Due to the wide range of malignance probability, category 4 is further divided into three subcategories: 4A, 4B and 4C, with 5–10%, 10–50% and 50–95% malignance probability, respectively. Category 5 is defined as highly suspected of malignancy, with more than 95% malignant probability (3). We focused our study on breast lesions classified as ACR BI-RADS US categories 4 or 5 because these lesions have a wide-ranging likelihood of malignancy (>5%) and were recommended for biopsy. Nevertheless, sonographic features for determining BI-RADS categories are generally based on the radiologist’s interpretation. Moreover, microcosmic features of images, such as gray matrix, texture features and so on, may not be identified by visual interpretation.

As is known to all, breast US is a sensitive imaging method for the diagnosis of BC. However, the ultrasound features of some atypical BC overlap with benign lesions (4), and thus may lead to miscategorization of benign cases into BI-RADS category 4 or 5 and unnecessary recommendation for biopsy. The positive predictive value of US-guided biopsies ranged from 19.5 to 42.7%, indicating that many patients did receive unnecessary biopsies, which is invasive and carries the risk of complications such as infection, hematoma and so on. Consequently, to make an exact diagnosis of both can prevent unnecessary operation, and at the same time prevent missed diagnosis of malignant tumor, which is of great significance for guiding clinical treatment. How to better distinguish the benign and malignancy of BI-RADS category 4 or 5 by non-invasive means is the burning issue at present.

In recent years, radiomics, a novel computer-aided technology that reflects the texture and morphological features of tumour by quantitatively analysing the grey values of medical images, has gradually received attention and application (5–8). Radiomics is a non-invasive reproducible low-cost technique which can extract many quantitative features from areas of interest of medical images through a computer algorithm. Most of the quantitative features extracted through computerized algorithms are beyond visual interpretation but may potentially be associated with important clinical outcomes, as they contain comprehensive information about tumor characteristics, such as tumor size, shape, strength, texture and so on (9–12). Radiomics features can be used to establish descriptive or predictive models, which is of great help to detection, diagnosis, treatment response prediction and prognosis evaluation in different types of cancer, and will eventually greatly improve clinical decision-making ability (6, 7, 13). Furthermore, nomograms have been widely used to predict medical prognosis and outcomes by combining multiple risk factors in different types of cancer. Of all the available models, a nomogram can provide an individualized, evidence-based, highly accurate risk estimation. Nomograms are easy to use and can facilitate management-related decision making.

Therefore, we aimed to explore radiomics score of US images of patients with BI-RADS category 4 or 5, and establish a nomogram of BC based on this and other clinical risk factors.

## Materials and methods

### Study population

**Figure 1:**
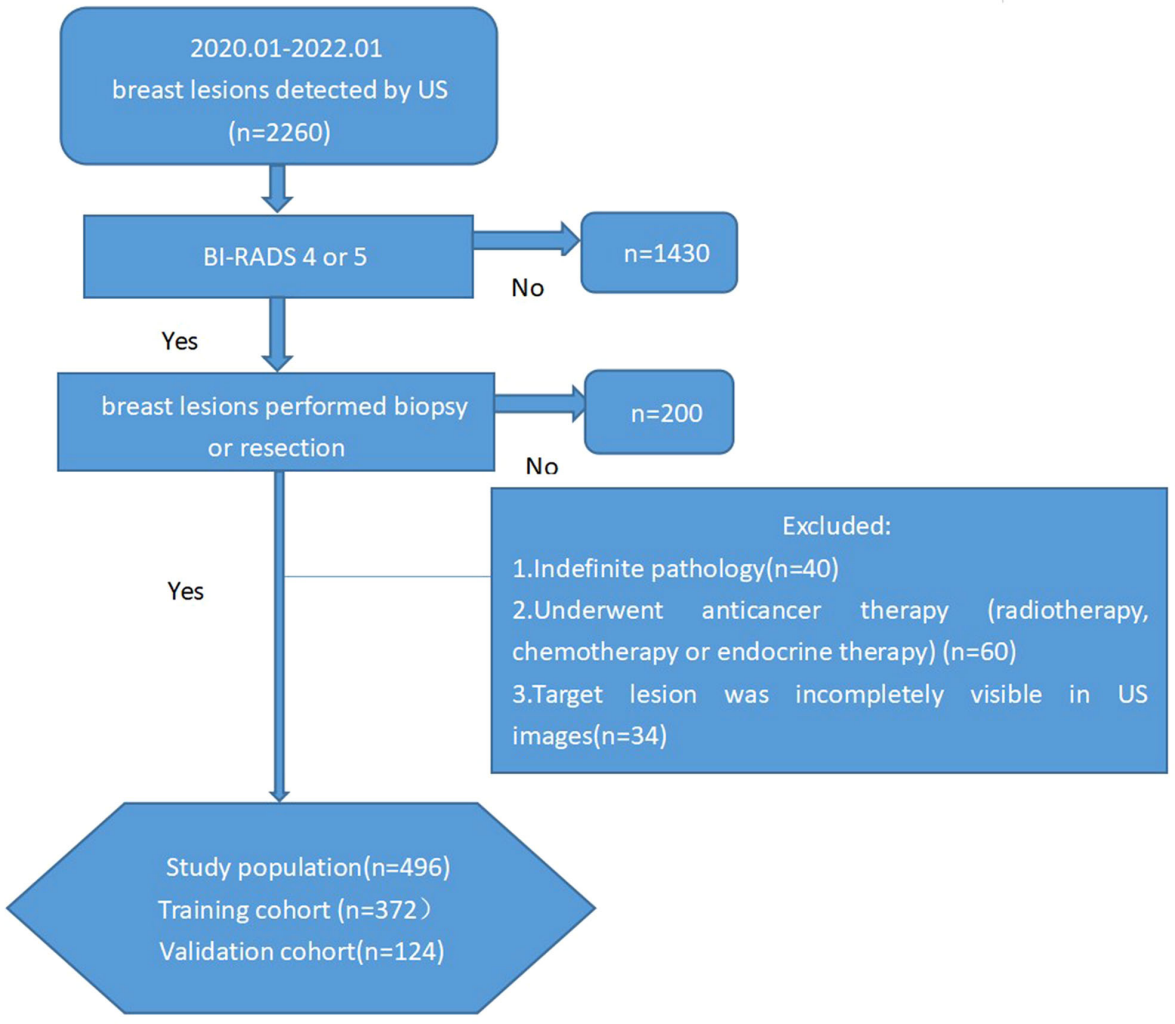
Flow chart of the study population enrolment.

The study was approved by the review committee of Fujian Provincial Hospital. Informed consent was waived because the present study is retrospective. Between January 2020 and January 2022,we collected female patients with US findings of breast lesions continuously. The following inclusion criteria were used: (1) a pathological result was available; (2) breast US was performed before biopsy or resection; (3) the target lesion was assigned as BI-RADS category 4A, 4B, 4C or 5 according to the second edition of the ACR BI-RADS US atlas. The following exclusion criteria were used: (1) the pathological result was indefinite;(2)images of tumors larger than 5 cm; (3) the patient had undergone anticancer therapy (chemotherapy, radiotherapy or endocrine therapy); or (4)the target lesion was incompletely visible on US. For patients with more than one breast lesion that was BI-RADS category 4 or 5, only the lesion with the highest BI-RADS category was included in the study to guarantee the statistical independence of each observation. Finally, a total of 496 lesions from 496 patients (mean age, 46.54 ± 11.75 years; range, 13 to 88 years) were included (**Figure 1**). Using the random sampling methods of SPSS version 20.0 (SPSS, Chicago, IL, USA), the data were split 75/25; 75% of the cases were assigned to the training group, which was used to establish the evaluation system, and 25% of the cases formed the validation group, which was used to test the accuracy of the model prediction.

### US and pathological examinations

US examinations were performed using an Philips EPIQ7 equipped with a L12-5 linear array probe. All of the breast lesions were examined and assessed by the same radiologist (L.JW) with over 30 years of experience of breast US examination. Imaging parameters were adjusted to optimally visualize the target lesion. The US characteristics were acquired in both transverse and longitudinal sections. The largest diameter of each lesion was recorded on the grayscale US images. Image preprocessing was performed on grayscale US images without considering whether imaging optimization technology, such as harmonics, SonoCT, and XRES, was used. We excluded images of tumors larger than 5 cm, as it is difficult to delineate the ROI properly if the tumor is not fully presented in a single plane. The US images were selected by two radiologists who had over 25 years of work of breast US examination and were responsible for the delineating of the ROI. The two radiologists were blinded to the patients’clinicopathological details.

In our study, breast lesions classified as BI-RADS category 4 or 5 were all recommended for biopsy. Pathological findings were confirmed by US-guided biopsy or surgical excision.US-guided biopsy was performed using 16-gauge or 18-gauge needle. More than three tissue samples were acquired and placed in formalin solution,followed by histopathological and immunohistochemical tests as per standard procedures (14). Patients with indefinite histological results were recommended for surgical excision.

### Definition of the ROI

**Figure 2:**
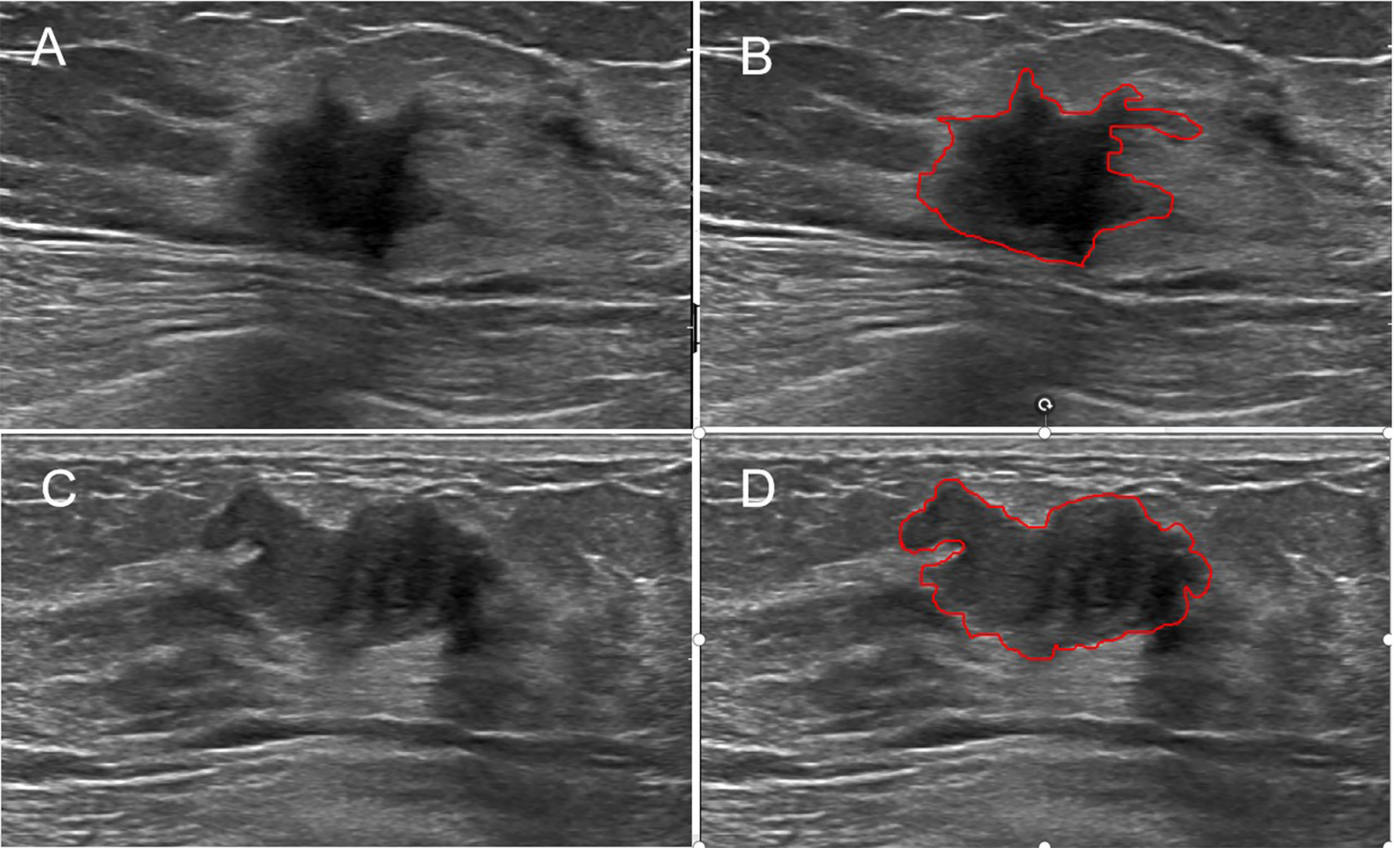
Examples of sketching ROIs on US images. The greyscale image **(A)** and the ROI **(B)** of a malignant lesion with the maximum section (radiomics score=−14.95). The greyscale image **(C)** and the ROI **(D)** of a benign lesion with the maximum section (radiomics score=−23.26).

Python software was used for radiomics analysis. The ROI was sketched along the edge of the breast tumor lesions on selected typical images of each patient and should include the whole lesion as far as possible. Finally the image of the whole tumor was obtained. Another radiologist (C.S) with 25 years of breast US experience, blinded to the final histopathological details, drew the ROIs using ITK-SNAP software (http://www.itksnap.Org/pmwiki//pmwiki.php) (**Figure 2**). To evaluate inter-observer variability, another radiologist (W.SS,specializing in breast US imaging for 25 years) drew the ROIs in 100 randomly selected lesions. In addition, to assess intraobserver reliability, C.S. then carried out the second delineation of ROIs from 100 randomly chosen images according to the same procedure one week later. The intra-observer agreement and inter-observer consistency of ROI portrayed by two radiologists were measured by the intraclass correlation coefficient (ICC). ICC > 0.75 is considered good consistency.

### Radiomics feature extraction and radiomics score

**Figure 3:**
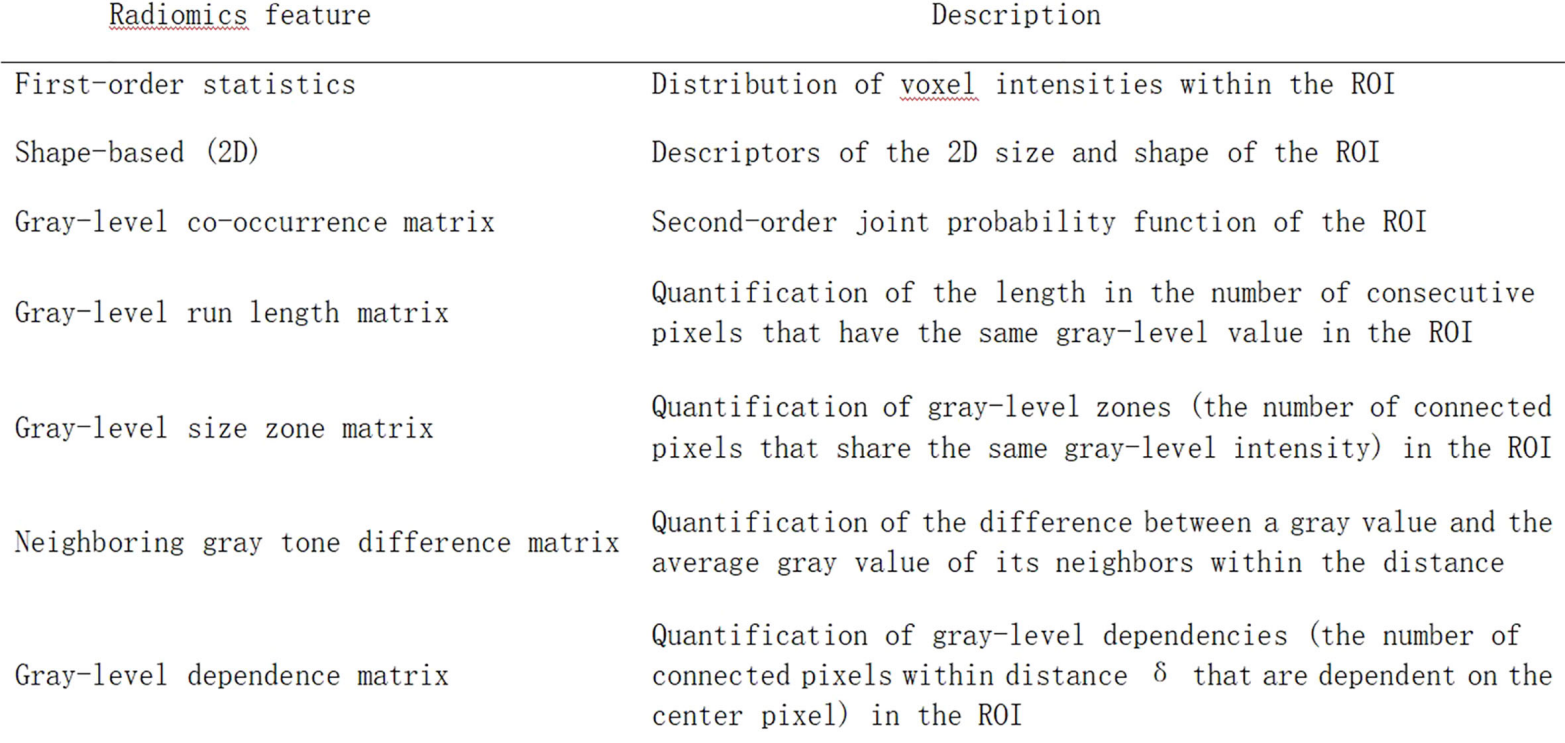
List of radiomics feature classes with descriptions from the PyRadiomics package.

We employed an open-source python package called"PyRadiomics" (15) to extract radiomics features from the US images, and all the results were collected in a form. These features included 5 neighboring gray tone difference matrix features, 16 gray-level run length matrix features, 16 gray-level size zone matrix features, 18 first-order features, 24 gray-level occurrence matrix features, and 14 gray-level dependence matrix features (**Figure 3**). Considering that multivariate analysis requires a 10:1 ratio of the number of patients and the number of covariates, logistic regression with the least absolute shrinkage and selection operator (LASSO) was chosen to extract radiomics features that might contribute to the prediction model (16). Then, a formula incorporating the selected features was developed to calculate the radiomics score.

### Model construction and validation

Univariate and multivariate logistic regression analyses were performed to analyse the significant factors associated with breast malignancy. The features were entered into statistical software to construct the prediction model on the basis of the multivariate logistic regression analysis. After construction, the prediction model was verified in the validation group. A calibration (i.e., consistency between the observed result frequencies and predicted probabilities) curve was plotted to explore the predictive accuracy of the nomogram. The specificity, sensitivity, positive likelihood ratio, negative likelihood ratio, positive predictive value, and negative predictive value were calculated to assess the diagnostic accuracy. Receiver operating characteristic (ROC) curves and the area under the curve (AUC) were generated to assess the discrimination performance of the prediction model. Decision curve analysis (DCA) was conducted to determine the clinical usefulness of the radiomics features by quantifying the net benefits at different threshold probabilities.

### Statistical analysis

R software (version 4.0.2) and SPSS 22.0 (Chicago, IL) were used to perform the statistical analysis. Python software was used for radiomics analysis. Student’s t-test was used to compare continuous variables with a normal distribution. The Mann-Whitney U test was used to compare continuous variables with an abnormal or unknown distribution. The χ² test was used to compare categorical variables. P values of less than 5% were considered statistically significant. The intra-observer agreement and inter-observer consistency of the two radiologists in ROI delineation were measured by the ICC, which was graded as very good (0.80 to 1.00), good(0.60 to 0.80), fair (0.40 to 0.60), moderate (0.20 to 0.40), or poor (< 0.20).R software was used to develop and assess the nomogram. The glmnet package was used for LASSO regression. The Hmisc package was used to perform the nomogram. The pROC package was used to plot the ROC curves and measure the AUCs. The difference in the AUC between the two groups was examined by the U test. The CalibrationCurves package was used for the calibration curves. The DecisionCurve package was used to plot the DCA.

## Results

### Baseline characteristics of the populations

**Table 1** shows the basic information of the research population. Breast malignancies occurred in 40.1% (149/372) and 44.3% (55/124) of the patients in the modeling and validation groups, respectively. There was no significant difference between the two groups for the presence of BC (P=0.589). No significant difference was detected between the two groups regarding the presence of BI-RADS category(p = 0.533), distribution of patient age (p = 0.064),largest lesion diameter (p = 0.38), or radiomics score(p = 0.827). **Table 2** displays the results of univariate and multivariate analyses for BC in the training group. The radiomics score,BI-RADS category, largest lesion diameter and patient age were demonstrated to be independent predictors of BC (P<0.001). The inter-observer consistency of the radiomics feature extraction between the two readers was substantial, with an ICC of 0.798 ± 0.021, and the intra-observer reliability reached good agreement, with an ICC of 0.829 ± 0.016.

**Table 1:** Basic information in the training and validation groups.

	Training group			Validation group		
	Benign	Malignant		Benign	Malignant	
	n = 223	n = 149	p	n = 69	n = 55	P
Age (years)	44.16 ± 11.80	52.15 ± 11.55	<0.001	41.75 ± 10.70	49.15 ± 11.80	<0.001
Diameter (mm)	13.33 ± 8.46	29.57 ± 13.98	<0.001	12.32 ± 6.72	26.53 ± 14.40	<0.001
BI-RADS			<0.001			<0.001
4A	154	24		55	13	
4B	59	29		13	15	
4C	10	45		1	13	
5	0	51		0	14	
Radiomics score	−18.44 ± 6.02	−3.57 ± 18.28	<0.001	−18.04 ± 7.99	−4.79 ± 15.94	<0.001

Diameter, Largest diameter of the target lesion.

Numerical data are presented as mean ± standard deviation, categorical data as numbers (n).

**Table 2:** Results of the univariate and multivariate analyses based on the development group.

	Univariate analysis		Multivariate analysis	
	OR (95% CI)	P	OR (95% CI)	P
Age (years)	1.06 (1.039,1.080)	<0.001	1.063 (1.031,1.095)	<0.001
Diameter (mm)	1.124 (1.097,1.153)	<0.001	1.060 (1.025,1.096)	0.001
BI-RADS				
4A	Ref.		Ref.	
4B	3.154 (1.699,5.854)	<0.001	2.134 (0.963,4.728)	0.062
4C	28.875 (12.858,64.845	<0.001	14.258 (5.035,40.378)	<0.001
5	NA	0.997	NA	0.996
Radiomics score	1.262 (1.195,1.334)	<0.001	1.209 (1.135,1.287)	<0.001

NA, values were not available.

### Radiomics score

**Figure 4:**
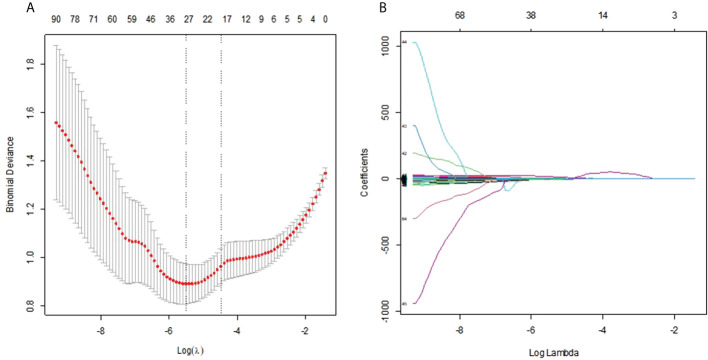
**(A)** Selection of the tuning parameter λ in the LASSO model *via* 10-fold cross-validation was based on the 1 standard error of the minimum criteria (the 1−SE criteria). The value of λ that gave the minimum average binomial deviance was used to select features. Dotted vertical lines were drawn at the optimal values using the minimum criteria and the 1−SE criteria. **(B)** LASSO coefficient profiles of the 112 radiomics features. A vertical line was drawn at the value selected using 10-fold cross-validation, where optimal result was 31 non-zero coefficients.

On the basis of the training group, 112 radiomics features were reduced to 31 potential predictors by the LASSO regression model (**Figures 4A, B**). The 31 features were included in the radiomics score formula as follows:

Radiomics score =−12.303134×diagnostics_Image.original_Mean−0.669834×diagnostics_Mask.original_VoxelNum+1.487857×original_shape_MajorAxisLength+0.384037×original_shape_Maximum2DDiameterRow−0.517837×original_shape_Sphericity−5.052795×original_shape_SurfaceArea+16.122379×original_shape_VoxelVolume-0.333529×original_firstorder_Kurtosis−0.081668*original_firstorder_Maximum−0.186704×original_firstorder_Minimum+0.703602×original_firstorder_RobustMeanAbsoluteDeviation−0.148799×original_glcm_Autocorrelation+0.083887×original_glcm_ClusterTendency−1.163571×original_glcm_DifferenceAverage−0.138501×original_glcm_DifferenceVariance+0.995198×original_glcm_Id−3.011644×original_glcm_Imc2−3.133431×original_glcm_JointAverage+0.331901×original_glcm_SumAverage+0.215143×original_gldm_DependenceNonUniformityNormalized−0.285513×original_gldm_LargeDependenceLowGrayLevelEmphasis−0.062460×original_glrlm_GrayLevelNonUniformity−0.654854×original_glrlm_GrayLevelVariance−0.369813×original_glrlm_LowGrayLevelRunEmphasis−2.467511×original_glrlm_RunEntropy+2.761×original_glszm_GrayLevelNonUniformity+1.013797×original_glszm_SizeZoneNonUniformityNormalized+0.104497×original_glszm_SmallAreaLowGrayLevelEmphasis+1.155782×original_ngtdm_Coarseness−1.747814×original_ngtdm_Complexity+0.09735×original_ngtdm_Strength

Malignant lesions had significantly higher scores than benign lesions in both groups (**Table 1**, both P<0.001).The radiomics signature showed good predictive efficacy, with an AUC of 0.886 in the training cohort and 0.868 in the validation cohort. The sensitivity of the radiomic signature was good, as high as 0.852 and 0.821 in the training and validation cohorts, and the specificity was also good, as high as 0.846 and 0.798 in the training and validation cohorts. The accuracies were 82% and 80% in the training and validation cohorts, respectively.

### Development and validation of the prediction model

**Figure 5:**
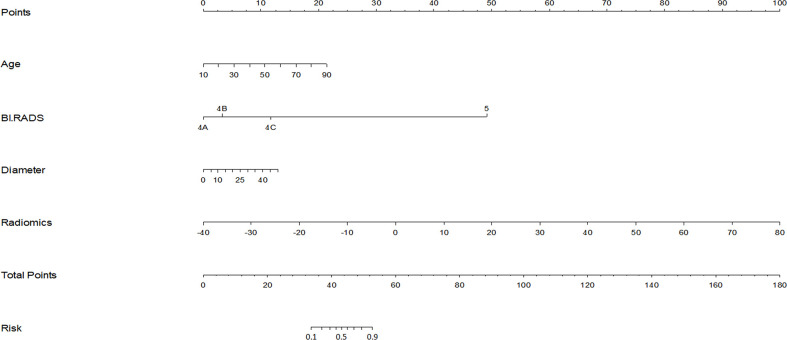
Nomogram for prediction of BC in BI-RADS US category 4 or 5 lesions. The different values for each variable corresponds to a point at the top of the graph, while the sum of the points for all the variables corresponds to a total point, draw a line from the total points to the bottom line is the probability of BC in BI-RADS US category 4 or 5 lesions.The AUC of the model constructed with radiomics score, patient age, lesion diameter identified by US and BI-RADS category involved was 0.956.

**Figure 6:**
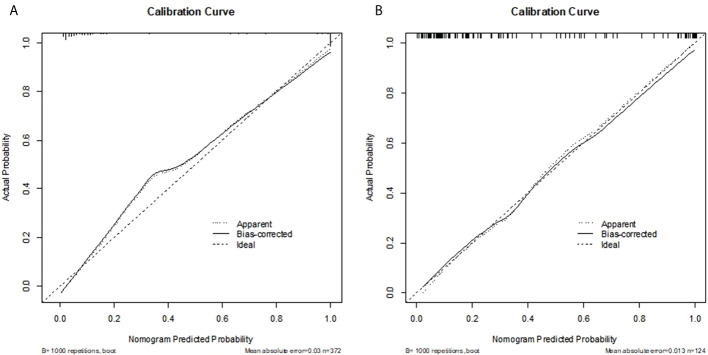
**(A, B)** Calibration curve of the nomogram for the development cohort and validation cohort. The X-axis represents the probability that nomogram predicted BC in BI-RADS US category 4 or 5 lesions, while Y-axis represents actual rate of it. The dotted line in the middle represents the perfect prediction, and the solid line represents the predictive power of the nomogram. The closer the solid line is to the dotted line, the better the predictive power of the model.

**Figure 7:**
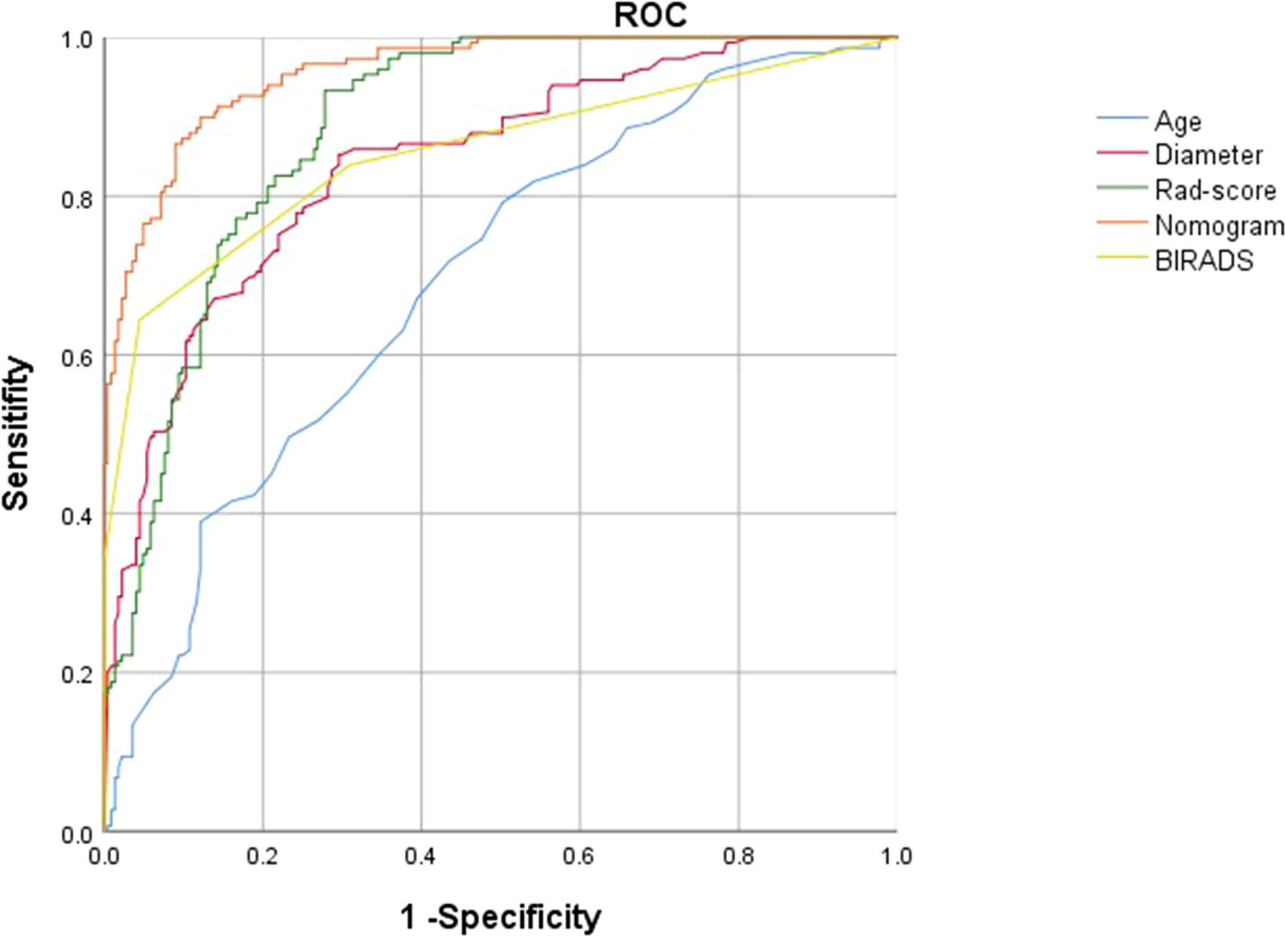
ROC curves of the radiomics score (green lines), BI-RADS category (yellow lines),patient age(blue lines),largest lesion diameter (red lines) and nomogram (orange lines) in the development groups.

Radiomics score, patient age, BI-RADS category and largest lesion diameter were all discovered as independent risks for BC in the multivariable logistic regression model (**Table 2**). We developed a nomogram based on a radiomic score, patient age, BI-RADS category and largest lesion diameter (**Figure 5**). Calibration curves of the full model in the training and validation groups were plotted to evaluate the agreement between the predicted probability of BC and actual results and are presented in **Figures 6A, B**. The bias curves for the training and validation cohorts are both close to the ideal line in the figures, and good consistency can be observed between the predictions and observations. The nomogram displayed an AUC of 0.956 (95% CI,[0.910, 0.972]) for predicting BC in the training cohort (**Figure 7**), and the sensitivity, specificity, and accuracy were 0.899,0.879, and 86%, respectively. In the validation cohort, it also displayed excellent prediction efficacy, with an AUC of 0.937 (95% CI, [0.893–0.965]), and the sensitivity, specificity, and accuracy were 0.886, 0.862, and 85%, respectively.

### Clinical use

**Figure 8:**
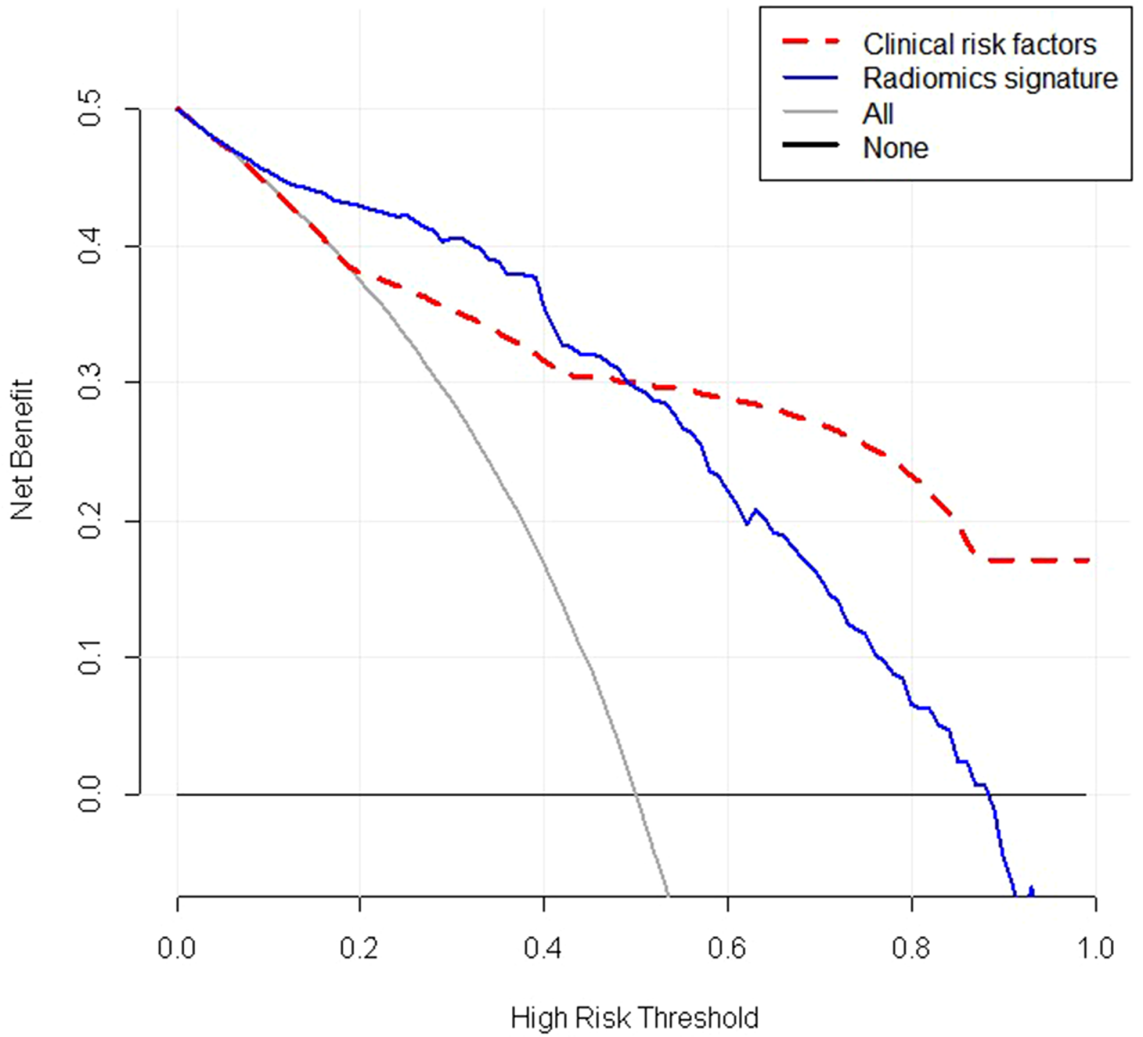
Decision curve analysis (DCA) of the radiomic signature(blue lines) and clinical risk factors(red lines). The y-axis represents the net benefit. The net benefit is determined by calculating the difference between the expected benefit and the expected harm related to each proposed model. The grey line represents the hypothesis that all lesions were malignant (the treat-all scheme). The black line represents the assumption that all lesions were benign (the treat-none scheme).As demonstrated in the curve, if the threshold probability was within a range from 0 to 0.9, the net benefit of using radiomics scores to predict malignancy added more benefit than either the treat-all scheme or the treatnone scheme. The net benefit of radiomics features were higher than that of clinical risk factors if the threshold probability was between 0 and 0.5. Radiomics signature: Radiomics score.

Decision Curve Analysis (DCA) evaluates and compares the clinical application value of imaging feature models and radiomics features by calculating the net benefit under different threshold probabilities (17). The decision curve analysis for the radiomics features and clinical risk factors were displayed in **Figure 8**. The decision curve analysis showed that when the threshold probability was within a range from 0 to 0.9, the net benefit of using radiomics features to predict BC added more benefit than either the treat-all scheme or the treatnone scheme. The net benefit of radiomics features were higher than that of clinical risk factors in the threshold probability within a range from 0 to 0.5,therefore,the radiomics features established in our study have high clinical application value.

## Discussion

In this research, we developed a nomogram based on a radiomics score and clinical characteristics to predict malignancy in breast lesions classified as BI-RADS US category 4 or 5,and it displayed excellent ability to predict malignancy in breast lesions with an AUC of 0.956 in the training cohort. In addition, we constructed a radiomics score, which also displayed good performance (AUC 0.886). Both of the nomogram and the radiomics score can assist clinicians to predict BC.

Radiomics is a rapidly developing computer-aided technology that converts medical imaging information into a series of data through computer algorithms, and then analyzes and models these data to provide potential non-invasive biomarkers for clinical decision making. Previous studies have shown that image microscopic characteristics are closely related to the microstructure and biological behavior of tumors (18–21). Due to the differences in time and space during tumor growth, pathological biopsy sometimes cannot represent the complete characteristics of tumor tissue. However, as radiomics signatures are composed of quantitative features extracted from images, they can describe tumor heterogeneity more comprehensively and noninvasively compared with subjective and qualitative features of tumor lesions (7, 22, 23). However, the association between biological behaviour and radiomics features is still complex (24). When biomarkers are selected from thousands of radiomics features, it is difficult to clearly clarify the relationship between radiomics features and biological behaviour. An effective method is to use radiomics techniques to establish multi-feature parameters for the estimation of results (25, 26). For the development of the radiomics signatures, 112 candidate radiomics features were reduced to 31 potential predictors by examining the predictor-outcome association by shrinking the regression coefficients with the LASSO method. This method not only surpasses the method of choosing predictors on the basis of the strength of their univariable association with outcome (27), but also enables the panel of selected features to be combined into a radiomics signature (28). Multimarker analyses that incorporate individual markers into marker panels have been embraced in recent studies (29–32), such as in the 21-gene assay that was identified and validated to spare the use of chemotherapy in certain groups of patients who have BC (31, 32). Similarly, in this study, we developed a radiomics signature that combine multiple individual imaging features extracted from the image of US, and the capability of the radiomics signature for estimating malignancy in breast lesions classified as BI-RADS US category 4 or 5 is impressing. The AUC, sensitivity, specificity, and accuracy were 0.868, 0.821,0.798, and 80% in the validation cohort.Thus, the noninvasive radiomics signature, which makes use of the images we already have for free, could serve as a more convenient biomarker for the prediction of BC.

Radiomics features are usually difficult to be interpreted and analyzed intuitively, but they can reflect the complexity and heterogeneity of tumor microenvironment. Take the features screened from the results as examples, sphericity is a measure of the roundness of the tumor area. The larger the value is, the closer it is to a perfect sphere; run entropy measures the randomness of run length and gray scale, and the higher the value, the more heterogeneous the mass; uniformity is the embodiment of the uniformity of image array, with a larger value meaning a greater uniformity (33). Although these features are beyond visual interpretation, they can be fully utilized by radiomics and provide considerable information for the diagnosis and prediction of diseases.

In our study, we found that many clinical characteristics were correlated with malignancy in breast lesions, including patient age, BI-RADS category and largest lesion diameter. As expected, the variables associated with BC in our study were quite similar to other studies (34–36). Taking into account the impact that clinical factors can have on BC, we developed a nomogram that incorporated radiomics signature and other clinical characteristics. We were encouraged that the nomogram showed excellent ability to evaluate BC with an AUC of 0.937 in the validation cohort. The nomogram is expected to reduce the difficulties faced by radiologists in the differential diagnosis of BC classified as BI-RADS US category 4 or 5, by greatly reducing the subjective guess of radiologist, and the negative influence of the uneven diagnosis levels of radiologists of varying seniority. With the nomogram, the diagnosis level tends to be uniform and improves as a whole.

There are some limitations to our study. First, this was a retrospective study, so inherent biases and variations were inevitable, and a prospective study should and would be conducted in the future for further verification. Second, the present study was a single-centre research study. In our study, although the performance of the nomogram has been evaluated by a validation cohort, additional validation at other centres will be necessary to assess the reliability of this prediction model. Third, breast lesions were ultimately assigned a category after analysing their sonographic features according to the second edition of the ACR BI-RADS US atlas. After verification, it has high diagnostic accuracy and was easy to use. Pure empirical diagnosis should be avoided in every case possible. We selected lesions classified as BI-RADS category 4 and 5, whose malignant risks were gradually increasing, and whose local malignant features were more prominent than benign features. In principle, it was better to include lesions classified as BI-RADS category 3, but it was not easy to obtain pathological results for them. Forth, US image features of lesions could be affected by pathological characteristics of that, the information about pathological characteristics of patients’ lesions was missing, and patients with indefinite pathology were excluded, selection bias might have occurred.

## Conclusions

We developed a radiomic signature and a nomogram based on a radiomic score, patient age, BI-RADS category and largest lesion diameter that can be used to identify BC in BI-RADS US category 4 or 5 lesions non-invasively, and the predictive performance is good. Both nomogram and radiomics score can be used as methods to assist radiologists and clinicians in predicting breast malignancy in BI-RADS US category 4 or 5 lesions.

## Data availability statement

The raw data supporting the conclusions of this article will be made available by the authors, without undue reservation.

## Ethics statement

The study was approved by the review board of Fujian Provincial Hospital. Informed consent was waived because the present study is retrospective.

## Author contributions

Z-LH and X-RP conceived the study, analyzed the data, and drafted the manuscript; Z-LH, SC, X-RP and S-SW helped critically revise the manuscript for important intellectual content. Z-LH, J-WL, J-CY helped collect data and design the study. All authors contributed to the article and approved the submitted version.

## Funding

Sponsored by Natural Science Fundation of Fujian Province (No.2020J011090).

## Acknowledgments

The authors are thankful to Fujian Provincial Hospital and Fujian Medical University for their management of our patient database. The authors are thankful to Song-Song Wu for helping critically revise the manuscript for important intellectual content and helping collect data and design the study.

## Conflict of interest

The authors declare that the research was conducted in the absence of any commercial or financial relationships that could be construed as a potential conflict of interest.

## Publisher’s note

All claims expressed in this article are solely those of the authors and do not necessarily represent those of their affiliated organizations, or those of the publisher, the editors and the reviewers. Any product that may be evaluated in this article, or claim that may be made by its manufacturer, is not guaranteed or endorsed by the publisher.

## Abbreviations

BI-RADS, Breast Imaging Reporting and Data System; US, ultrasound; BC, breast cancer; WHO, World Health Organization; AUC, Area under the curve; GLCM, Gray level co-occurrence matrix; GLRLM, Gray level run length matrix; GLSZM, Gray level size zone matrix; ROC, Receiver operating characteristic; ROI, regions of interest.
